# In the Digital Era, Is Community Outrage a Feasible Proxy Indicator of Emotional Epidemiology? The Case of Meningococcal Disease in Sardinia, Italy

**DOI:** 10.3390/ijerph15071512

**Published:** 2018-07-18

**Authors:** Marco Dettori, Benedetto Arru, Antonio Azara, Andrea Piana, Gavino Mariotti, Maria Veronica Camerada, Paola Stefanelli, Giovanni Rezza, Paolo Castiglia

**Affiliations:** 1 Department of Medical, Surgical and Experimental Sciences, University of Sassari, Via Padre Manzella 4, 07100 Sassari, Italy; madettori@uniss.it (M.D.); benedettoarru@gmail.com (B.A.); azara@uniss.it (A.A.); piana@uniss.it (A.P.); 2 Department of Humanities and Social Sciences, University of Sassari, Via Roma 151, 07100 Sassari, Italy; mariotti@uniss.it (G.M.); vcamerada@uniss.it (M.V.C.); 3 National Health Institute, Viale Regina Elena 299, 00161 Rome, Italy; paola.stefanelli@iss.it (P.S.); giovanni.rezza@iss.it (G.R.)

**Keywords:** community outrage, digital epidemiology, emotional epidemiology, meningococcal disease, vaccine

## Abstract

The aims of this study were (i) to evaluate the relationship between official data on invasive meningococcal disease cases in Sardinia and the reporting of the cases by a regional online newspaper and (ii) to identify indicators useful for understanding the community outrage related to health events. Cases of meningococcal disease, selected from articles published between 1999 and 2016 on a regional newspaper database, were compared to those reported to the Infectious Disease Information Service. In order to evaluate the equality of the two distribution records, the Kolgomorov Smirnov test for two samples was applied. A community outrage indicator was obtained by calculating the number of published articles for each case of meningococcal disease identified. The outrage indicator was evaluated in comparison with other phenomena: drinking water supply limitation and domestic accidents. Overall, 2724 articles on meningitis/sepsis referring to 89 cases related to meningococcal disease were considered. Significant differences between the distribution of cases officially reported and those found in the newspaper (combined K-S = 0.39; *p* = 0.08) were not observed. The meningococcal disease outrage indicator showed an average of seven items per case. Comparing the meningococcal disease outrage indicator with those regarding the limitation of drinking water supplies and domestic accidents, a different risk perception by the reference media was found, with the highest outrage for meningococcal disease. The present study supports the role played by emotional factors as behavioral determinants in emerging threats to public health. The analysis of the data allowed us to highlight that the proposed outrage indicator could be a feasible proxy of emotional epidemiology. Finally, data confirm that meningitis is perceived as a highly outrageous health threat.

## 1. Introduction

In order to better define social and communication phenomena related to meningitis, to which the media have given a great deal of attention in recent months in Italy, it is certainly worth referring to the emotional epidemiology approach [1].

Despite data from the National Surveillance System of Invasive Bacterial Diseases showing a substantial stability in the trend of cases due to *Neisseria meningitidis*, *Haemophilus influenzae* and even *Streptococcus pneumoniae* over the last two years [2], the growing risk perception and anxiety of the population led to an increased access to health services, which is known as “rush to vaccinate”. In particular, on January 2017, people of all ages swamped vaccination services, asking not only for the meningococcal C, but also for meningococcal B vaccine [3], which is now recommended for schoolchildren in Italy [4]. This vaccine was specifically indicated for newborns, based on a countrywide epidemiological investigation performed in 2014 [5], and consequently recommended by the Lifetime Immunization Schedule of several Italian scientific societies [6].

Actually, this national phenomenon of panic can be attributed to an increase of severe cases of invasive meningococcal disease sustained by a hyper-virulent strain of *Neisseria meningitidis* serogroup C, which affected the central area of Tuscany in 2015–2016 [7,8].

Rare diseases with high case-fatality rates and epidemic potential, such as meningitis, recall ghosts of the past and activate collective mechanisms of defense, whose dynamics are completely consistent with the transmissibility of the same infectious agents. The media, for their part, are a strong catalyst of the above-mentioned emotional reactions, reporting with increasing frequency all suspected cases. In this context, the efforts of health authorities may hardly reassure the population [9].

Such emotional dynamics have been well described by Peter Sandman in his “hazard vs. outrage” theory. Sandman has shown that the perception of risk is not only a function of “hazard” (magnitude × probability) but also, and above all, it is closely related to the entire emotional experience of fear, anger and concern, which he has termed “outrage” [10].

On this basis, knowing the outrage level related to each health event becomes pivotal in order to respond with proper communication strategies (i.e., precaution advocacy, crisis communication, outrage management), which must be calibrated according to the perceived level of risk.

As the media usually publish what is of interest to the population, the number of published articles may represent a proxy for community outrage.

It is now clear how important the role played by mass media through proper communication is. Moreover, it should also be considered that, because of the current globalization of information, health data can be available almost in real-time on the network, intercepting (or even anticipating) the report of outbreaks with a sensitivity approaching official data through epidemiological surveillance [11,12,13]. The study of these mechanisms has revolutionized Public Health [14] and has given rise to a new discipline called digital epidemiology [15].

The Italian island of Sardinia has already proven to be well trained in epidemiological studies due to its insularity, which preserves the region from interferences caused by the territorial contiguity [16]. Therefore, it can represent an excellent exercise for the reported social and epidemiological dynamics.

The purpose of this study is (i) to evaluate the relationship between official statistics of the invasive meningococcal disease in Sardinia and the reporting of cases by a regional online newspaper, and (ii) to identify indicators useful for understanding the community outrage related to health events.

## 2. Materials and Methods

A retrospective analysis of all the articles published between 1999 and 2016, concerning invasive meningococcal disease, was performed by consulting the online database of a regional newspaper. The article selection procedure used four keywords: meningitis, meningococcus, sepsis, and fulminant. Counting the cases required two investigators to independently read the selected articles, following the criteria of “specificity”, selecting cases attributable to meningococcus; “exhaustiveness”, recruiting as many cases as possible; “mutual exclusivity”, in order to avoid counting the same cases, especially when described in different instances in the newspaper.

Datasets used and/or analyzed during the current study are available from the corresponding author on reasonable request. Data obtained were firstly compared to those reported in the Infectious Disease Information Service tables (SIMI) [2]. In order to evaluate the equality of the two distribution records, a Kolgomorov Smirnov test for two samples was performed. Data were analyzed with STATA_11 software [17]. The set significance level was 0.05.

Secondly, a meningococcal disease outrage indicator (MD-OI) was obtained by calculating the number of published articles for each case identified. The consistency of the MD-OI was verified by consulting articles published in the year 2010 (which was the first useful year to access all the online databases) and in 2016 (which offered the most up-to-date data) by 22 local newspapers from different Italian regions. The research followed the aforementioned criteria of article selection and reading procedures.

In order to better appreciate the role played by community outrage in risk perception, the MD-OI was compared to other phenomena characterized, unlike meningococcal disease, by a high incidence in the island: drinking water supply limitation owing to public notices, and domestic accidents.

Thus, a retrospective search of all the articles concerning the limitation of drinking water supplies and domestic accidents was performed, using the online search engine of the newspaper previously consulted for the meningococcal disease.

Regarding drinking water supply, the observation was limited to a three-year (2013–2015) timeframe, excluding the years before 2013, which fell into a period when the ministerial derogation instrument was still in force [18].

On the contrary, as far as domestic accidents were concerned, the study was limited to the year 2014, which offered the most up-to-date official statistics [19].

Keywords used for the selection process were, for the first topic, drinking water and ordinance, while for the second, they were accident and domestic. The same methodology was followed, wherein the two investigators independently read all the articles retrieved, according to the criteria of specificity, exhaustiveness, and mutual exclusivity.

Then, the number of drinking water limitations and domestic accidents which occurred in Sardinia were obtained consulting a recent paper [20] and the official 2014 incidence reporting [19], respectively.

Outrage indicators for drinking water (DW-OI) and domestic accident (DA-OI) were properly calculated by the ratio between the number of published articles and the events found.

In order to compare the community outrage indicators, they were evaluated contextually considering the incidence of each phenomenon. As far as meningococcal disease and domestic accidents are concerned, statistics from official sources were used to estimate their incidence [2,19,21]. Conversely, because of the lack of official statistics, the incidence estimation of drinking water supply limitation was retrieved from the paper of Dettori et al. [20]. In particular, it was possible to calculate an incidence estimate, given the relationship between the number of observed alerts and the “equivalent aqueduct days” (number of water dispensing days during the three-year period observed, multiplied by the 32 operating aqueduct schemes in the territory) [22].

## 3. Results

During the period 1999–2016, 2724 articles on meningitis/sepsis published by the regional newspaper were examined. In particular, 637 (23.4%) referred to 89 cases related to meningococcal disease. Non-meningococcal cases reported by the media were most commonly viral or pneumococcal diseases, followed by other cases attributable to unspecified bacteria. During the same period, SIMI reported 145 cases of meningococcal disease, with an incidence of 0.005 × 1000 in the Island.

**Figure 1:**
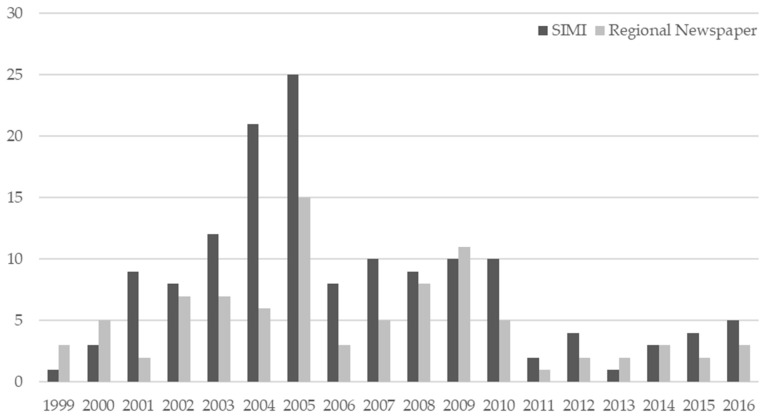
Number of cases of meningococcal disease according to official sources and regional media distributed per year of observation. In order to evaluate the outrage in meningococcal meningitis/sepsis. SIMI: Infectious Disease Information Service tables.

**Figure 2:**
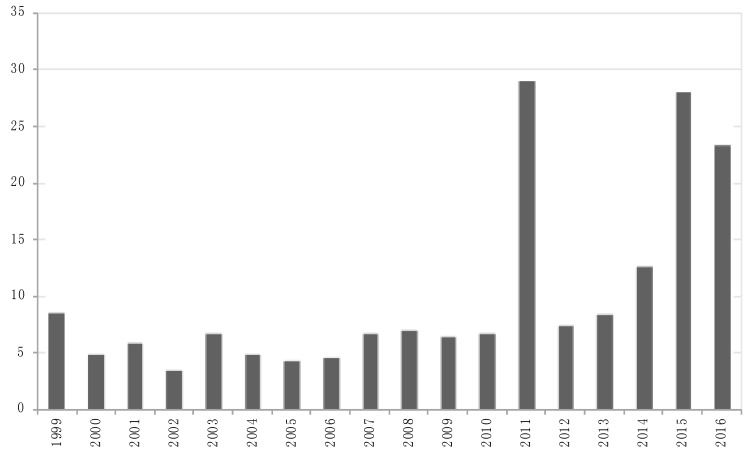
Number of articles published per year by the newspaper for a single case of attested meningococcal disease.

Figure 1 shows the distribution of meningococcal disease cases, according to SIMI and the regional media, per year of observation. Overall, there were no significant differences in the distribution of cases between those officially reported and those found in the newspaper (combined K-S = 0.39; *p* = 0.08). Moreover, there was a similarity between the two sources, especially in the years with fewer cases, with increasing sensitivity of media coverage in recent years. Figure 2 shows the distribution per year of the number of published articles per single case of disease.

The MD-OI showed an almost constant level over the period, with a median of seven items per case. In 2011, there was an increasing number of articles published per case. Actually, an extraordinary event due to legal action against a medical team, who cared for an 8-month-old child who died of a fulminant meningitis, has led to a large number of articles being published daily. However, a growing trend from 2012 is appreciable up to 2015.

Additionally, the MD-OI indicator consistency was verified by consulting 22 local newspapers of different Italian regions, comparing the number of published articles per case of meningococcal disease with those observed during the same period in Sardinia. Overall, 585 and 2032 articles were selected in the year 2010 and in 2016, respectively; of them, 23.9% (140/585) and 62.1% (1261/2032) referred to a total of 22 meningococcal disease cases reported in the year 2010 and to the 115 cases reported 2016. The average of articles per case were 6.36 (95% CI: 4.50–8.22) in 2010 and 22.64 (95% CI: 9.74–35.4) in 2016, which was not significantly different from that found in Sardinia at the same period (6.80 and 23.33).

Thus, the MD-OI was compared with outrage indicators obtained for the limitation of drinking water supplies (DW-OI) and domestic accidents (DA-OI).

The database consultation and the subsequent selection of articles by the criteria of specificity, exhaustiveness and mutual exclusivity returned, respectively, 3553 and 1620 articles for drinking water and 360 and 25 articles for domestic accidents. At the same time, 560 limitations of drinking water supply for the three years considered and 23,460 domestic accidents in 2014 were found.

The ratio between the number of articles and recorded cases allowed us to evaluate the DW-OI (median value 2.6) and DA-OI (median value 0.01).

**Figure 3:**
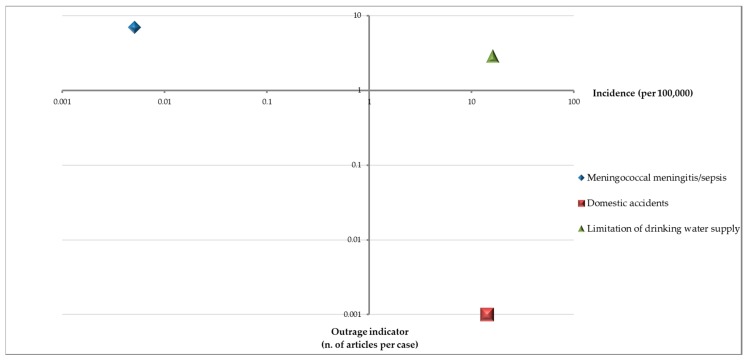
Bivariate distribution of outrage indicators and incidence of each phenomenon.

Actually, the estimated incidence rates for the water supply limitations in the three-year period 2013–2015 and for the domestic accidents in 2014 were 15.98 × 1000 and 14.1 × 1000, respectively. Figure 3 shows the bivariate distribution of incidence and outrage indicators for the three phenomena studied.

The risk perception was different, since outrage resulted very high for meningitis, although these were low incidence events. On the contrary, domestic accidents had a high incidence but a lower outrage. Finally, the regional issues in supply of water, due to recurrent drought and a widespread qualitative decline, was confirmed to have a high incidence and a medium-high outrage [23,24,25].

## 4. Discussion

Although only one newspaper was considered in the study, the results are representative of the entire regional area, owing to the presence of specific provincial sections in the format. Furthermore, consulting articles from different Italian newspapers allowed us to verify the consistency of our outrage indicator. In particular, the value observed in the regional newspaper was similar to those obtained from the national ones during both 2010 and 2016, two years which were characterized by very different outrage levels due to an outbreak which occurred in Tuscany in 2016, which generated a public alert. Therefore, we may consider the MD-OI indicator to be robust in relation to outrage variability.

Overall, as far as meningococcal disease is concerned, we have not noticed significant differences between the newspaper-reported cases and the official notifications. This suggests that, in low-density housing areas such as in Sardinia, it is possible to assess trends of low-incidence but highly emotional diseases such as meningitis or meningococcal sepsis (which can be extremely lethal or have severe consequences) by using mass media reports, which have a sensitivity close to those of official statistics and are updated in real-time.

We also observed that the sensitivity of the newspaper in detecting disease cases greatly increased during the last period, producing also an increase in the outrage indicator.

The outrage increase was likely due to the outbreak which occurred in Tuscany in 2015–2016, where the number of meningococcal diseases increased due to the circulation of a hyper-virulent strain of meningococcus C [8]. Additionally, the Tuscany event led to a “rush to vaccinate”, a real assault on immunization centres in all Italian regions.

In practice, on the one hand, the media are able to intercept and report every detail of each case of disease with high sensitivity and with real-time updates; on the other hand, the same media often transmit information to the population without taking into account the epidemiological evidence and real numbers which characterize the disease annually.

Another noteworthy consideration is the “news adaptation effect” observed in the years 2002–2004 (Figure 1): at that time, while the official sources reported a significant increase in meningococcal disease cases, the number of articles published on the newspaper was limited.

Finally, it is important to note what happened in 2011, when outrage increased following the death of an 8-month-old girl affected by meningitis. In that case, suspicious medical malpractice was likely to drastically increase the outrage, becoming an important determinant of emotional epidemiology.

Before drawing conclusions, we would like to underline, as a possible limit or strength of our study, that we decided not to use programming languages or other similar products for web scraping, preferring the support of two independent observers, which obtained data from the search engines of the online newspapers databases in order to accurately evaluate the number of single events specifically related to meningococcal invasive disease.

## 5. Conclusions

The present study confirms the role played by emotional factors in emerging public health phenomena and allows us, for the first time, to reflect on its community outrage dependence. Analyzing data captured by an online newspaper on meningococcal diseases, limitation of water supplies, and domestic accidents enabled us to highlight the quality of information and the strong correspondence between digital and official data, highlighting how outrage indicators could be a feasible proxy in evaluating emotional epidemiology. Finally, meningitis was observed to be among the most outrageous health phenomena. Public health communication should include outrage management for low incidence events with high emotional impact, such as meningitis [25,26]; precaution advocacy for high incidence events with a low perception of risk, such as domestic accidents [27]; and adequate risk communication for high incidence events with medium-high outrage, such as public water restriction alerts [28].

In conclusion, since citizens’ concern is not a function of a real risk, it is desirable that the media themselves cooperate with public health authorities to keep risk perception at the real level of the hazard.
